# Non-invasive fast assessment of hepatic injury through computed tomography imaging with renal-clearable Bi-DTPA dimeglumine

**DOI:** 10.1093/rb/rbae118

**Published:** 2024-10-03

**Authors:** Li Ma, Jinbin Pan, Gang Shu, Haiyan Pan, Jingang Li, Dong Li, Shaokai Sun

**Affiliations:** Department of Radiology, Tianjin Key Laboratory of Functional Imaging, Tianjin Medical University General Hospital, Tianjin 300052, China; Department of Radiology, Tianjin Key Laboratory of Functional Imaging, Tianjin Medical University General Hospital, Tianjin 300052, China; Department of Radiology, The Second Hospital of Tianjin Medical University, Tianjin 300211, China; Department of Radiology, Tianjin Key Laboratory of Functional Imaging, Tianjin Medical University General Hospital, Tianjin 300052, China; Department of medical technology, Taishan Vocational College of Nursing, Shandong 271000, China; Department of Radiology, Tianjin Key Laboratory of Functional Imaging, Tianjin Medical University General Hospital, Tianjin 300052, China; School of Medical Imaging, Tianjin Key Laboratory of Functional Imaging, Tianjin Medical University, Tianjin 300203, China

**Keywords:** bismuth, computed tomography, hepatic ischemia–reperfusion, renal-clearable

## Abstract

Enhanced computed tomography (CT) imaging with iodinated imaging probes is widely utilized for the diagnosis and evaluation of various liver diseases. However, these iodine-based imaging probes face intractable limitations including allergic reactions and contraindications. Herein, we propose the utilization of renal-clearable iodine-free bismuth chelate (Bi-DTPA dimeglumine) for the non-invasive fast assessment of hepatic ischemia–reperfusion injury (HIRI) *via* CT imaging for the first time. Bi-DTPA dimeglumine offers several advantages such as simple synthesis, no purification requirement, a yield approaching 100%, large-scale production capability (laboratory synthesis > 100 g), excellent biocompatibility and superior CT imaging performance. In a normal rat model, the administration of Bi-DTPA dimeglumine resulted in a significant 63.79% increase in liver CT value within a very short time period (30 s). Furthermore, in a HIRI rat model, Bi-DTPA dimeglumine enabled the rapid differentiation between healthy and injured areas based on the notable disparity in liver CT values as early as 15 min post-reperfusion, which showed a strong correlation with the histopathological analysis results. Additionally, Bi-DTPA dimeglumine can be almost eliminated from the body *via* the kidneys within 24 h. As an inherently advantageous alternative to iodinated imaging probes, Bi-DTPA dimeglumine exhibits promising prospects for application in liver disease diagnosis.

## Graphical abstract

**Graphical Abstract:**
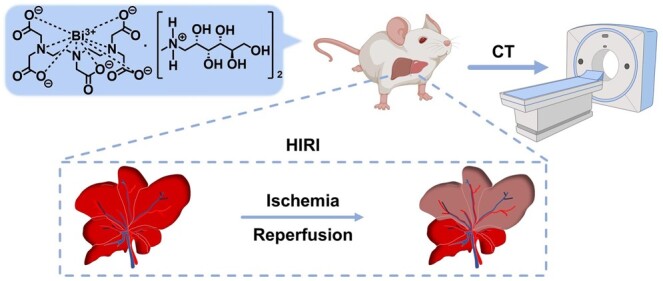

## Introduction

Hepatic ischemia–reperfusion injury (HIRI) refers to liver damage caused when the liver undergoes a period of inadequate blood supply followed by the restoration of blood flow [1, 2]. The mechanism of injury involves adenosine triphosphate (ATP) reduction, reactive oxygen species generation, inflammatory cells activation and adhesion molecules upregulation, which may lead to liver cell apoptosis and necrosis, impacting liver function and potentially causing final liver failure [3–5]. HIRI is a prevalent and challenging-to-avoid common complication in various clinical events, including liver resection, transplantation, trauma or hemorrhagic shock [6, 7]. HIRI can contribute to up to 10% of transplant failures and may increase the incidence of acute and chronic rejection, directly affecting patient prognosis and elevating morbidity and mortality rates [8, 9]. Therefore, the precise and fast diagnosis of HIRI holds significant medical value.

Currently, common methods for detecting and assessing HIRI in clinical practice include blood biochemistry analysis, tissue biopsy and imaging examinations. Despite offering comprehensive information about overall liver function, blood biochemical analysis lacks the capability to provide detailed anatomical insights into liver damage [10]. While tissue biopsy is widely considered the gold standard for identifying liver injury, it is limited to localized information and is invasive, preventing real-time dynamic tracking of lesion progression [11]. Imaging examinations not only provide visual information regarding anatomical structures [12] but also offer a safe, comprehensive and real-time assessment of HIRI [13–17]. Among various imaging techniques, CT imaging, with the advantages of high imaging speed, spatial resolution, cost-effectiveness and powerful post-processing capabilities, has become a powerful and frequently used diagnostic tool in clinical settings for assessing HIRI [18, 19]. Currently, iodine-based radiopaque agents constitute the most prevalent class of CT contrast media employed in clinical settings. Notwithstanding, the relatively low atomic number (*Z* = 53) and K-edge energy (33.2 keV) of iodine constrain the X-ray attenuation efficacy of iodine-based contrast agents [20, 21]. Besides, iodine-based contrast agents are facing increasing concerns related to iodine allergies and contraindications for individuals with thyroid conditions, which significantly limit their widespread use [22].

So far, a range of high atomic number elements have been used in the manufacture of CT imaging probes, such as ytterbium (Yb, *Z* = 70), hafnium (Hf, *Z* = 72), tantalum (Ta, *Z* = 73), gold (Au, *Z* = 79), bismuth (Bi, *Z* = 83), etc. [20, 21, 23–26]. For example, the Ta_2_O_5_@CuS NPs, Au NPs and Hf-doped carbon dots have been explored in the CT imaging diagnosis of orthotopic hepatic tumors [27–29], and Fe_3_O_4_-Au NPs as CT-MR dual-modality contrast agents for the accurate detection of alcoholic liver and liver cirrhosis [30]. Bi, with its exceptionally high atomic number and K-edge energy (90.5 keV), exhibits unparalleled X-ray attenuation capabilities across a broad spectrum of X-ray energies [26]. Furthermore, a number of medications based on bismuth have been extensively utilized to treat inflammation and gastrointestinal infections due to their excellent biocompatibility [31]. Additionally, bismuth is the most affordable heavy metal suitable for CT imaging. Therefore, bismuth is one of the optimal elements for constructing high sensitivity CT imaging probes [32–34]. In recent decades, various bismuth-based imaging probes such as Bi [35–44], Bi_2_S_3_ [45–54], Bi_2_Se_3_ [55, 56], Bi_2_O_3_ [57, 58], BiF_3_ [59], BiOI [60–63] and BiOCl [64] have been developed for CT imaging. However, a prominent characteristic of these nanoparticles is the high susceptibility to extensive phagocytosis by mononuclear phagocyte systems, leading to prolonged accumulation in the liver and spleen. The degradation mechanism is intricate, making it challenging to metabolize and excrete them from the body, posing significant safety concerns. Particularly concerning the diagnosis of liver damage, the substantial accumulation of nanoparticles in the liver increases the risk of exacerbating liver injury [65, 66]. In addition to Bi-based nanoprobes, our group has proposed Bi chelates as CT imaging probes [67–69], which possess outstanding renal clearance capability. Since different diseases have distinct histological characteristics, the uptake, accumulation and metabolism of imaging probes vary accordingly. Therefore, it is of great significance to investigate the diagnostic capabilities of high-performance imaging probes for various diseases. Although non-iodine-based imaging probes, especially Bi-based imaging probes, have made remarkable progress, they have never been studied or applied in the diagnosis of HIRI.

**Scheme 1.**
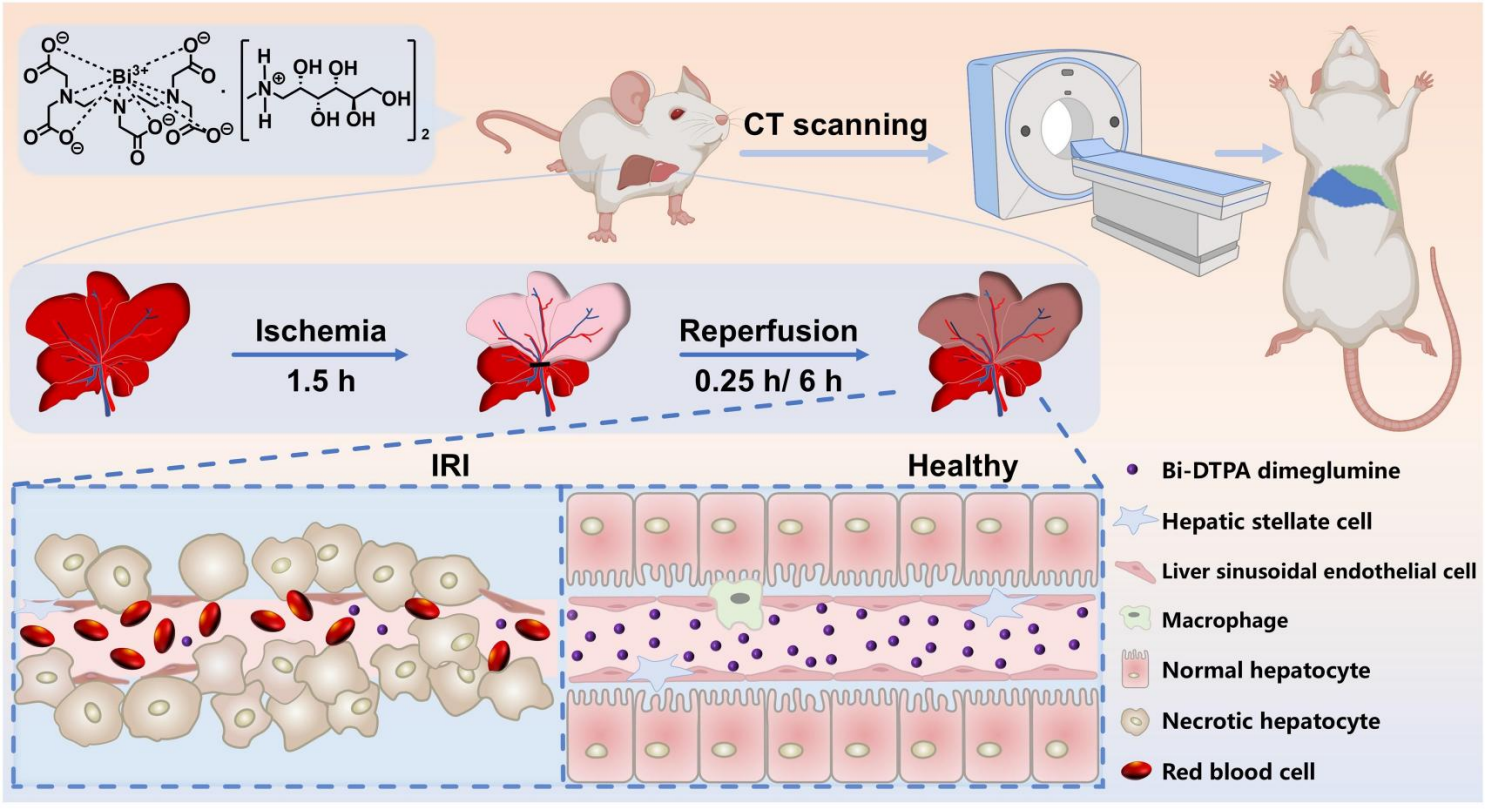
Schematic illustration of non-invasive fast assessment hepatic injury through computed tomography using Bi-DTPA dimeglumine.

We demonstrate the inaugural non-invasive rapid evaluation of HIRI *via* CT imaging utilizing renal-clearable Bi-DTPA dimeglumine (Scheme 1). Bi-DTPA dimeglumine, prepared through a straightforward purification-free method, exhibits excellent water solubility, high yield and the capability for large-scale production (up to hundreds of grams in laboratory synthesis), along with superior CT imaging performance. Systematic *in vivo* and *in vitro* evaluations confirm the biocompatibility of Bi-DTPA dimeglumine, showing rapid renal clearance without liver retention. In normal rats, injection of the imaging probe resulted in a 63.79% increase in liver CT values in a short time (30 s). In HIRI rats, CT imaging based on Bi-DTPA dimeglumine accurately and rapidly delineates the anatomical location and contours of liver damage with different degrees, which correlates well with the results of histopathological analysis. This study presents a highly sensitive and biocompatible CT imaging technique for diagnosing HIRI, free from concerns related to iodine allergies and contraindication to iodine.

## Materials and methods

### Materials 

All reagents employed were of at least analytical grade and were utilized without any further purification. Wahaha Group Co., Ltd. (Hangzhou, China) provided the ultra-pure water for the experiment. Aladdin Biochemical Technology Co., Ltd. (Shanghai, China) supplied Bi_2_O_3_, DTPA, N-methyl-_D_-glucamine, xylenol orange (XO) and methyl thiazolyl tetrazolium (MTT). Fetal bovine serum (FBS) was acquired from Bailing Biotechnology Co., Ltd. (Lanzhou, China). The medium [Dulbecco’s Modified Eagle Medium (DMEM)] was obtained from Thermo Scientific Co., Ltd. (Suzhou, China). Concord Technology Co., Ltd. (Tianjin, China) supplied the dimethyl sulfoxide (DMSO).

### Synthesis of Bi-DTPA dimeglumine

After vigorously shaking a mixture of 50 mmol Bi_2_O_3_ and 100 mmol DTPA in 400 mL H_2_O at a temperature of 85°C for a duration of 2 h, the dark yellow suspension progressively became transparent over time, signifying the production of Bi-DTPA. After cooling to 50°C, N-methyl-_D_-glucamine (200 mmol) was introduced and stirred for an additional hour. The obtained Bi-DTPA dimeglumine solution can be used directly after appropriate dilution or freeze-dried into powder for various characterizations and biological applications.

### Characterizations of Bi-DTPA dimeglumine

Fourier transform infrared (FT-IR) spectroscopy was performed using a Nicolet IS10 spectrometer (Nicolet, USA), with the spectra recorded across the wavenumber range of 4000-400 cm^−1^. Pure potassium bromide (KBr) was utilized as the background reference. Ultraviolet-visible-near-infrared (UV-vis-NIR) absorption spectra of Bi-DTPA were measured with UV-3600 PLUS spectrophotometer (Shimadzu, Japan). High-resolution electrospray ionization mass spectra (HRMS) were acquired using an LTQ-Orbitrap Mass Spectrometer (Thermo Fisher, USA). The ^1^H NMR spectra of Bi-DTPA and Bi-DTPA dimeglumine were recorded on an Avance II 400 MHz spectrometer (Bruker, Switzerland), with chemical shifts reported in parts per million (ppm) using D_2_O as the solvent. The concentration of Bi element was determined by inductively coupled plasma optical emission spectrometer (ICP-OES, Agilent 5800, USA).

### *In vitro* CT imaging

To evaluate the CT imaging capability, various concentrations of Bi-DTPA dimeglumine and iohexol (0, 12.5, 25, 50, 100 and 200 mM radiopaque elements) were prepared. CT imaging was performed using a dual-energy CT scanner (Somatom Force, Siemens Healthineers, Erlangen, Germany). Scanning parameters were set to the following: tube voltage, 70-150 kV (10 kV increments); adaptive tube current; slice thickness, 0.5 mm; and field of view, 256 mm × 256 mm.

### Stability

To assess the stability of Bi-DTPA dimeglumine, it was dissolved in various solvents at a final concentration of 0.1 M, including deionized water, DMEM, normal saline (NS), PBS (pH 7.4, 10 mM) and FBS. Photographs were taken of these solutions on days 0, 7, 14 and 30. The chemical structural stability of Bi-DTPA was assessed using 0.2% XO solution. Free Bi^3+^ can react with XO to form a red-colored chelate, and the leakage of Bi^3+^ can be determined by using a microplate reader (Synergy HTX, BioTek, USA).

### *In vitro* cytotoxicity assay

The normal mouse hepatocyte cells (AML12) and mouse embryonic fibroblast cells (3T3) were cultured in DMEM cell medium supplemented with 10% fetal bovine serum and 1% streptomycin-penicillin and incubated in a constant temperature incubator at 37°C and 5% carbon dioxide. The cytotoxicity of Bi-DTPA dimeglumine to AML12 cells and 3T3 cells was determined by standard MTT assay. AML12 cells and 3T3 cells were incubated into 96-well plates with a density of 1 × 10^4^ cells per well and cell medium containing 200 µL per well were cultured in incubators for 24 h. Following the removal of the stale medium, the cells were washed with PBS, and then treated with 200 µL of fresh medium containing various concentrations of Bi-DTPA dimeglumine (0, 1.25, 2.5, 5, 10 and 20 mM), and incubated for 24 h. Then, the old medium was discarded, and each well was washed with PBS, followed by the addition of 200 µL of fresh medium containing MTT (0.25 mg/mL). After 4 h, the MTT-containing medium was sucked out, and 120 µL of DMSO was added to each well to dissolve the formazan purple precipitate generated by viable cells. After gentle shaking for 5 min, the absorbance at 490 nm per well was measured using a microplate reader to calculate the cell survival rate. Each experiment was conducted in triplicate.

### Hemolysis assay

Animal experiments were performed in accordance with the guidelines for laboratory animal care and use at Tianjin Medical University General Hospital, with approval from the Institutional Animal Care and Use Committee (IRB2022-DW-76). Male SD rats, sourced from SPF Biotechnology Co., Ltd. (Beijing, China), were maintained in a controlled environment with a standardized 12-h light-dark cycle and allowed unrestricted access to food and water. Fresh blood samples (2 mL) were collected from SD rats, placed in anticoagulant tubes, mixed with 3 ml saline solution and allowed to stand for 1 h. Following centrifugation (3000 rpm, 5 min), the erythrocyte sediment was washed three times with normal saline solution before being suspended in 4 mL of normal saline solution. Subsequently, red cell suspensions were incubated with various concentrations of Bi-DTPA dimeglumine (10, 25, 50 and 100 mM), saline solution as a negative control group and pure water as a positive control group. After a 2-h incubation period, plates were centrifuged at 3000 rpm for 5 min. Hemolysis was initially observed through photographs taken during this process. The liquid above the sediment was then transferred into a plate and its ability to absorb light at a wavelength of 541 nm was measured using a device for analysing samples in microplates. The rate of hemolysis (HR) = [(Abs(sample) – Abs(negative control))/(Abs(positive control) – Abs(negative control))] × 100%.

### *In vivo* toxicity of Bi-DTPA dimeglumine

The *in vivo* biocompatibility of Bi-DTPA dimeglumine was evaluated through weight monitoring, blood biochemical analysis, histopathological assessment and blood sodium test. The experimental group of rats received intravenous administration of Bi-DTPA dimeglumine (1.6 mmol Bi/kg, *n* = 3), while the control group received an equivalent volume of saline (*n* = 3). Over a period of 14 days, body weights were documented every alternate day. Blood samples were collected at four distinct time intervals (initial, day 1, day 7 and day 14) for the purpose of biochemical analysis. The assessment involved evaluating liver function markers such as alanine aminotransferase (ALT), aspartate aminotransferase (AST), alkaline phosphatase (ALP), serum albumin (ALB) and total protein levels (TP). Furthermore, kidney function indicators including serum creatinine (CREA), uric acid levels (UA) and blood urea nitrogen levels were also measured. Additionally, major organs including heart, liver, spleen, lung and kidney were collected from different groups (control, 1 day and 14 days) for hematoxylin-eosin (HE) staining. To investigate the effect of Bi-DTPA dimeglumine on serum sodium levels, venous blood samples from rats (*n* = 3) were collected before and at various time points (5 min, 30 min, 2 h and 24 h) after the administration of Bi-DTPA dimeglumine, and were used to monitor changes in sodium levels using an electrolyte analyser (IMS-972, XiLaiHeng, Shenzhen, China).

In order to assess the impact of Bi-DTPA dimeglumine on HIRI, a total of 36 SD rats (240-260 g) were utilized to establish models of HIRI. One group was subjected to 1.5 h of ischemia, followed by a reperfusion period lasting 0.25 h, whereas the other group underwent 1.5 h of ischemia and then experienced reperfusion for a duration of 6 h. Each group was randomly divided into six subgroups, with three rats in each subgroup. Three subgroups received immediate injections of Bi-DTPA dimeglumine after modeling, whereas the remaining three subgroups did not receive any injections post-modeling. Subsequently, blood samples and liver specimens were collected at different time points: immediately after modeling, one day after modeling and seven days after modeling to evaluate liver injury. Blood biochemical analysis was employed to assess the effect of Bi-DTPA dimeglumine on liver injury.

### *In vivo* CT imaging of normal rats

The rats were anesthetized with isoflurane inhalation (RWD Life Science, Shenzhen, China) at a concentration of 1-1.5% and a flow rate of 0.4-1 L/min during CT imaging. Male SD rats (240-260 g) were fasted for 12 h, and then CT imaging was carried out before the intravenous injection of Bi-DTPA dimeglumine (1.6 mmol Bi/kg). Liver CT images were captured at various time points (2 s, 30 s, 60 s, 120 s, 5 min, 10 min, 2 h and 24 h). The control group received an injection of iohexol solution (1.6 mmol I/kg), and CT images were collected under the same parameters and time points. Subsequently, CT signal analysis was conducted on the major organs, including the liver, kidneys and bladder.

### *In vivo* metabolic behavior of Bi-DTPA dimeglumine

To evaluate the circulation half-life of Bi-DTPA dimeglumine, rats (*n* = 3) were injected with Bi-DTPA dimeglumine (1.6 mmol Bi/kg) *via* the tail vein. Blood samples were collected from the jugular vein at different time points (5, 10, 30, 60, 120 and 240 min). The samples were then dissolved in concentrated nitric acid at 70°C for 3 h and diluted to a suitable concentration. Bi levels were measured using ICP-OES. To investigate the biodistribution of Bi-DTPA dimeglumine, rats (*n* = 3) were injected with Bi-DTPA dimeglumine (1.6 mmol Bi/kg) *via* the tail vein, and subsequently euthanized at pre-injection and post-injection time points (10 min, 24 h and 7 d) to collect major organs (heart, liver, spleen, lungs and kidneys). After weighing, the organs were dissolved in concentrated nitric acid at 70°C for 4 h and then diluted to a suitable concentration. The Bi content was quantified using ICP-OES.

### *In vivo* CT imaging of HIRI rats

To establish a 70% HIRI model, SD rats were anesthetized with isoflurane (at a concentration of 1-1.5% with a flow rate of 0.6-1 L/min) and placed in a supine position. After routine disinfection of the rats, the median incision of the abdomen was made, the duodenal ligament was separated and the first hepatic hilum was exposed. The blood vessels did not to be clamped in the sham surgery group, while the left middle hepatic artery and portal vein were clamped by vascular forceps in the surgery group, causing a rapid color change in the left and median lobe of the liver from reddish-brown to light brown. Subsequently, the incision was covered with moist gauze, and the animal was placed on a heating pad to prevent hypothermia. After 1.5 h of ischemia, the vessel clamp was removed, and the left and median liver lobes began to return to reddish-brown color within a few seconds. Another 1 mL sterile saline was injected into the rat's abdomen to replace fluid loss during surgery, and the abdomen was closed by sutures during reperfusion. The HIRI group was divided into two groups, and CT scan was performed at 0.25 and 6 h after reperfusion. The HIRI rats were first subjected to a blank control scan, followed by intravenous injection of Bi-DTPA dimeglumine or iohexol (1.6 mmol radiopaque elements/kg) through high pressure injector at a rate of 0.5 mL/s. After the completion of the CT scanning (10 min), the blood of rats was collected from the inner canthus of the rats immediately to assess blood biochemical parameters, and the left and right liver lobes were dissected for pathological analysis. The rats in the sham group were injected with Bi-DTPA dimeglumine and iohexol (1.6 mmol radiopaque elements/kg), and CT imaging, blood collection and liver lobes dissection were carried out according to the afore-mentioned procedure.

### Liver CT image analysis

The main parameters for assessing CT image quality are the signal-to-noise ratio (SNR) and ΔSNR. The regions of interest (ROIs) were delineated according to the guidelines of including as much enhanced area as possible while minimizing vascular and motion artifacts for CT images of healthy and injured liver. The CT values, SNR and ΔSNR within the ROIs were acquired to evaluate the imaging efficacy of Bi-DTPA dimeglumine and iohexol, with SNR and ΔSNR calculated using the following formulas:

$$\begin{array}{l} SNR=\frac{CT\;value}{SD\;n(noise)}\\ \Delta SNR=\frac{CT\;value(healthy\;liver)-CT\;value(injured\;liver)}{SD\;n(noise)} \end{array}$$

## Results and discussion

### Synthesis and characterization of Bi-DTPA dimeglumine

**Figure 1.**
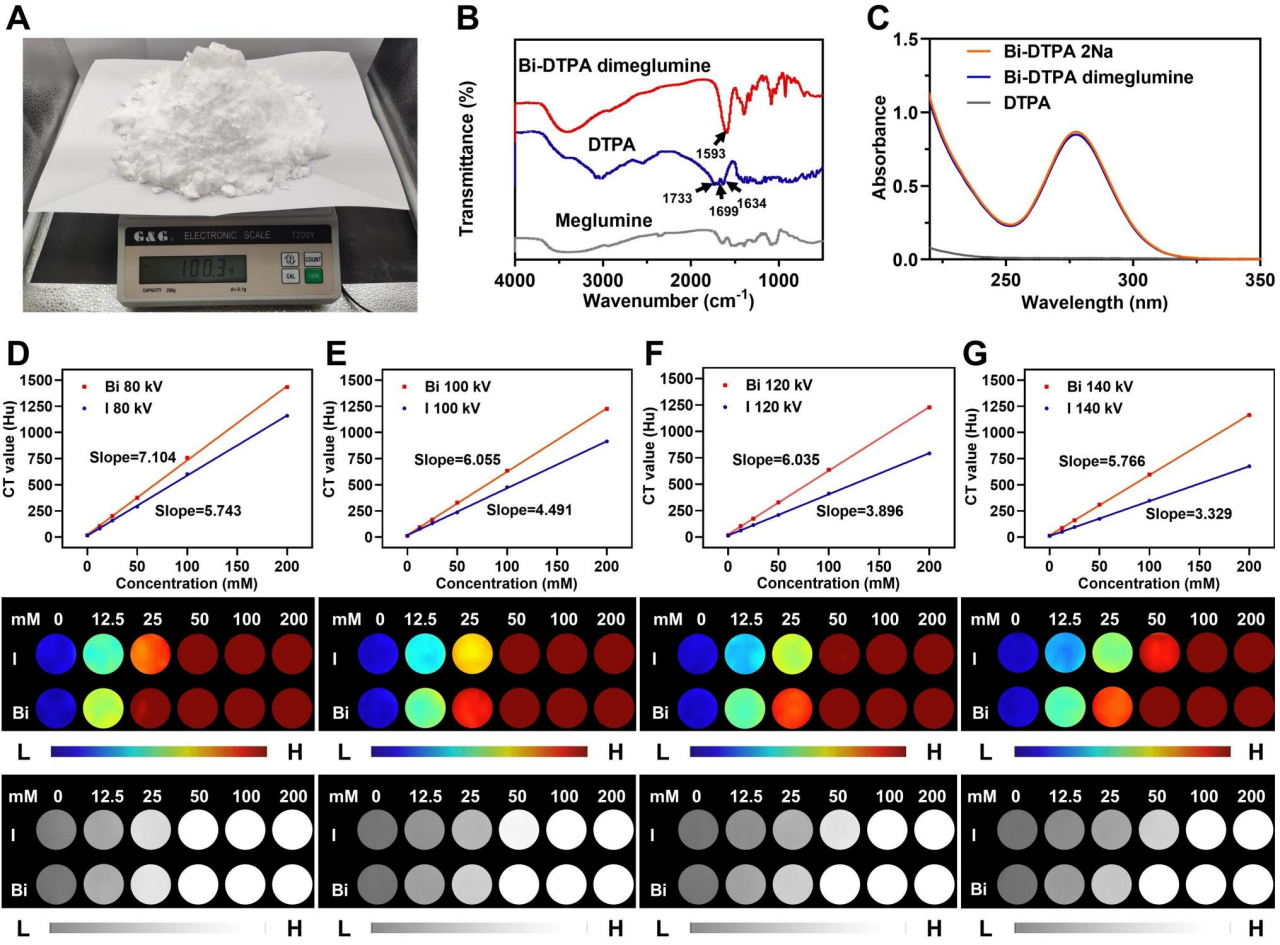
Characterization and *in vitro* CT phantom study of Bi-DTPA dimeglumine. (**A**) Large-scale synthesis of Bi-DTPA dimeglumine. (**B**) FT-IR spectra of meglumine, DTPA and Bi-DTPA dimeglumine. (**C**) UV-vis-NIR absorption spectra of DTPA and Bi-DTPA. Hu curves and CT images of Bi-DTPA dimeglumine and iohexol at different concentrations (0, 12.5, 25, 50, 100 and 200 mM Bi/I) at (**D**) 80 kV, (**E**) 100 kV, (**F**) 120 kV and (**G**) 140 kV.

To synthesize Bi-DTPA dimeglumine, a mixture of Bi_2_O_3_ and DTPA was dissolved in water and subjected to heating at 85°C for 2 h. This resulted in the formation of a clear solution of Bi-DTPA through an acid-base neutralization and coordination reaction. Subsequently, N-methyl-_D_-glucamine was introduced to adjust the pH level to 7.4. This exceptionally simple synthesis process enables the rapid production of hundreds of grams of Bi-DTPA dimeglumine in a single batch in laboratory settings (Figure 1A). The purity of the product was evaluated by determining the content of free Bi^3^^+^ through dimethyl phenol orange titration (Supplementary Figure S1). Free Bi^3+^ can react with XO to form a stable red-colored complex with a characteristic absorption at 570 nm (Supplementary Figure S1A). As shown in Supplementary Figure S1B, no significant red compound Bi-XO was detected in the mixture of Bi-DTPA and XO, which demonstrated that the purity of Bi-DTPA was nearly 100%. Even on the 15th day, free Bi^3+^ was still undetectable, indicating that Bi^3+^ does not leak from Bi-DTPA dimeglumine.

The molecular structure of Bi-DTPA dimeglumine was determined by HRMS, and the peak at *m*/*z* 598.0849 in HRMS was ascribed to Bi-DTPA structure ([M+H]^-^: calculated for C_14_H_19_BiN_3_O_10_: 598.0880). ^1^H NMR spectra further confirmed the structure of Bi-DTPA dimeglumine [^1^H NMR (400 MHz, D_2_O) *δ* 4.27 (s, 2H), 4.13-4.01 (m, 4H), 3.97-3.67 (m, 14H), 3.66-3.56 (m, 4H), 3.41-3.25 (m, 4H), 3.22-3.09 (m, 4H), 3.04 (t, *J* = 12.3 Hz, 2H), 2.72 (s, 6H)] (Supplementary Figure S2). The FT-IR analysis revealed the presence of stretching vibrations related to -COOH- and -CONH- functional groups in DTPA. These vibrations exhibited peaks at specific wavenumbers of approximately 1733, 1699, and 1634 cm^−1^ (Figure 1B). Interestingly, upon formation of the Bi-DTPA dimeglumine complex, these stretching vibrations vanished while a distinct O-C-O (carboxylate) vibration peak emerged at around 1593 cm^−1^ in the FT-IR spectrum. Furthermore, an absorption peak characteristic to this complex was observed near approximately 280 nm wavelength range according to the absorption spectra data (Figure 1C). To investigate the enduring colloidal stability, a solution of Bi-DTPA dimeglumine (0.1 M) was prepared in various aqueous media including water, DMEM, normal saline (NS), PBS (pH 7.4, 10 mM) and FBS. Remarkably, no precipitation was observed throughout a duration of 30 days, which indicated Bi-DTPA dimeglumine showed excellent solubility and stability in each medium (Supplementary Figure S3).

### *In vitro* CT imaging

The X-ray absorption capacity of Bi-DTPA dimeglumine and iohexol was investigated by comparing the image brightness and CT values at various concentrations (Figure 1D–G and Supplementary Figure S4). As the molar concentration of the radiopaque element increased, both Bi-DTPA dimeglumine and iohexol exhibited a gradual increase in image brightness (Figure 1D–G and Supplementary Figure S4). Furthermore, at equivalent molar concentrations of the radiopaque element, Bi-DTPA dimeglumine consistently demonstrated higher image brightness compared to iohexol. Additionally, for both Bi-DTPA dimeglumine and iohexol, the CT values displayed a concentration-dependent linear increase at constant voltage settings. Notably, as the tube voltage rose from 80 to 140 kV, the disparity in slope between them became more pronounced. For instance, at a clinically common X-ray tube voltage of 120 kV, the fit line slope for Bi-DTPA dimeglumine was measured as 6.035, which is significantly higher than that of iohexol (3.896) (Figure 1F). These findings suggested that Bi-DTPA dimeglumine possessed superior X-ray absorption capabilities.

### Biotoxicity assessment *in vivo* and *in vitro*

**Figure 2.**
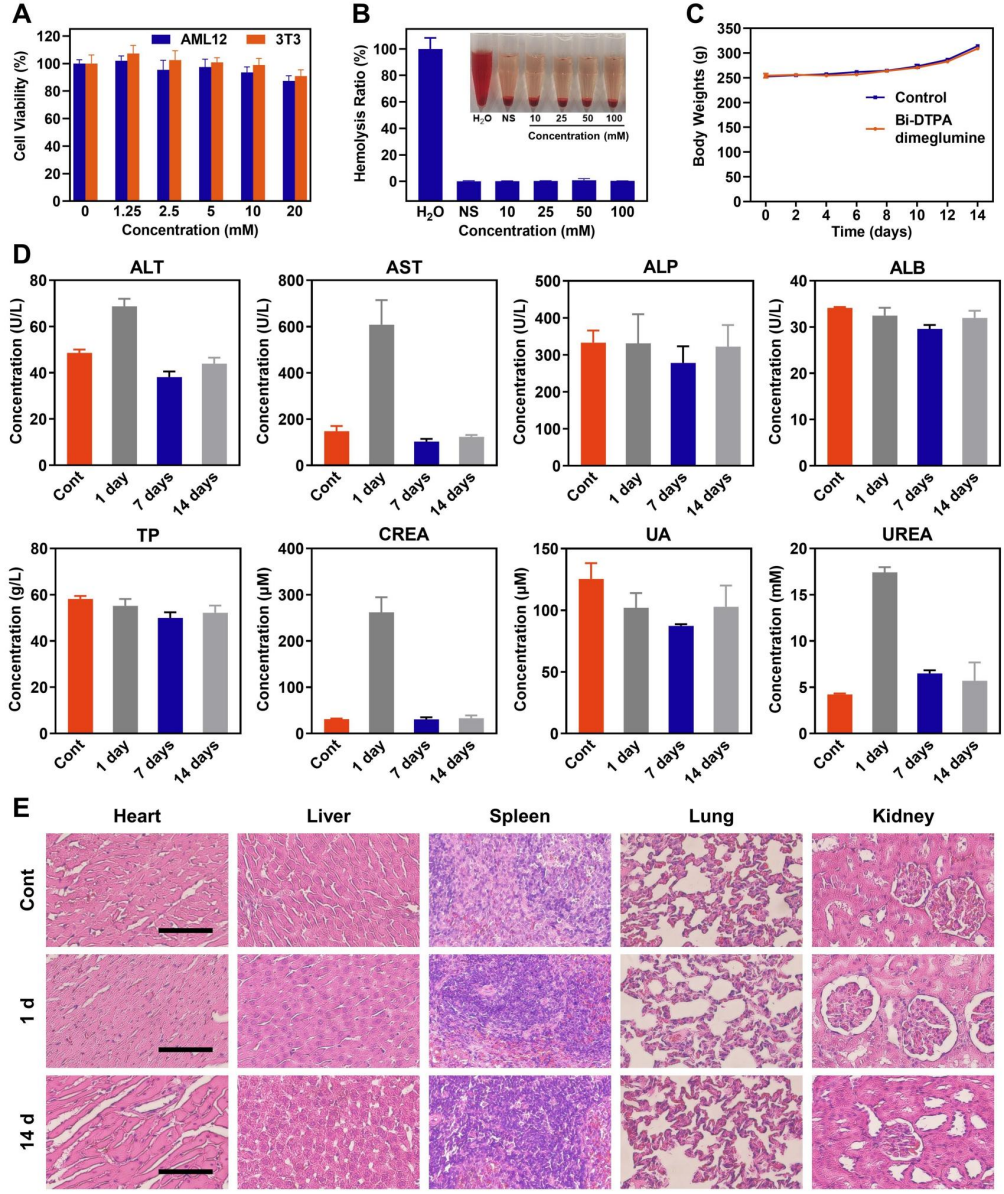
The toxicity of Bi-DTPA dimeglumine *in vitro* and *in vivo*. (**A**) Cellular viabilities of AML12 cells and 3T3 cells after incubation of different concentrations of Bi-DTPA dimeglumine. (**B**) Hemolysis ratio of Bi-DTPA dimeglumine with different concentrations (0, NS, 10, 25, 50 and 100 mM). (**C**) Body weights monitoring of normal rats after intravenous injection of Bi-DTPA dimeglumine (1.6 mmol Bi/kg) for 14 days. (**D**) Biochemical analysis of rats including liver and kidney functions in 0, 1, 7 and 14 days. (**E**) HE staining images of the major organs (heart, liver, spleen, lung and kidney) in 0, 1 and 14 days. Scale bar: 100 μm.

The standard MTT assay was utilized to evaluate the cytotoxicity of Bi-DTPA dimeglumine. Various concentrations of Bi-DTPA dimeglumine (0, 1.25, 2.5, 5, 10 and 20 mM) were administered to both 3T3 cells and AML12 cells for a duration of 24 h. When the concentration of Bi-DTPA dimeglumine reached 20 mM, the cell proliferation rate remained above 80% (Figure 2A), indicating that Bi-DTPA dimeglumine, as a small-molecule imaging probe, exhibits low cytotoxicity. To evaluate the hemocompatibility, Bi-DTPA dimeglumine with different concentrations were incubated with red blood cells for 1 h. Based on visual examination and absorbance measurement at 541 nm, it was observed that the hemolysis rate of Bi-DTPA dimeglumine was comparable to that of physiological saline, even when its concentration reached as high as 100 mM (Figure 2B), demonstrating the good hemocompatibility of Bi-DTPA dimeglumine. Subsequently, normal rats were intravenously injected with Bi-DTPA dimeglumine for long-term *in vivo* toxicity assessment. The weight of rats injected with Bi-DTPA dimeglumine or normal saline was recorded every two days, and the results showed no significant difference between the experimental and control groups within 14 days (Figure 2C). In comparison to the control group, no significant differences were observed in blood biochemical indicators associated with liver and kidney function, including ALT, AST, ALP, TP, ALB, CREA, UA and UREA in both the 7-day and 14-day groups (Figure 2D). It should be noted that major liver and kidney function indicators (ALT, AST, UREA, CREA) significantly increased after one day but returned to normal levels after seven days due to the introduction of the imaging probe resulted in transient liver and kidney dysfunction. Compared to other imaging techniques, CT imaging has relatively low sensitivity (imaging probe, CT: 10^−3^ M; MRI: 10^−3^-10^−5^ M; ultrasound imaging: 10^−6^-10^−9^ M; PET: 10^−11^-10^−12^ M) [70], often necessitating the use of large doses of imaging agents [24]. Therefore, the use of CT contrast agents may result in temporary side effect [71]. In addition, the sodium test results indicated that administration of Bi-DTPA dimeglumine did not induce hypernatremia in rats (Supplementary Figure S5). Histopathological results of the rats at 1 day and 14 days after the injection of Bi-DTPA dimeglumine showed no significant tissue injury or necrosis in sensitive organs (heart, liver, spleen, lung and kidney) compared to the control group (Figure 2E).

To investigate whether the introduction of the imaging probe would exacerbate liver injury, *in vivo* toxicity assessments were conducted on rats with different degrees of liver injury induced by HIRI (Supplementary Figure S6). Blood biochemical analysis results indicated that kidney function indicators (UREA and CREA) exhibited a transient increase after 1 day (Supplementary Figure S6B and E), but returned to normal levels after 7 days. The elevated liver function indicators showed negligible increase in the HIRI rats (Supplementary Figure S6C and F) after the injection of Bi-DTPA dimeglumine. These results demonstrated the favorable biocompatibility of Bi-DTPA dimeglumine both *in vitro* and *in vivo*.

### *In vivo* CT imaging of normal rats

**Figure 3.**
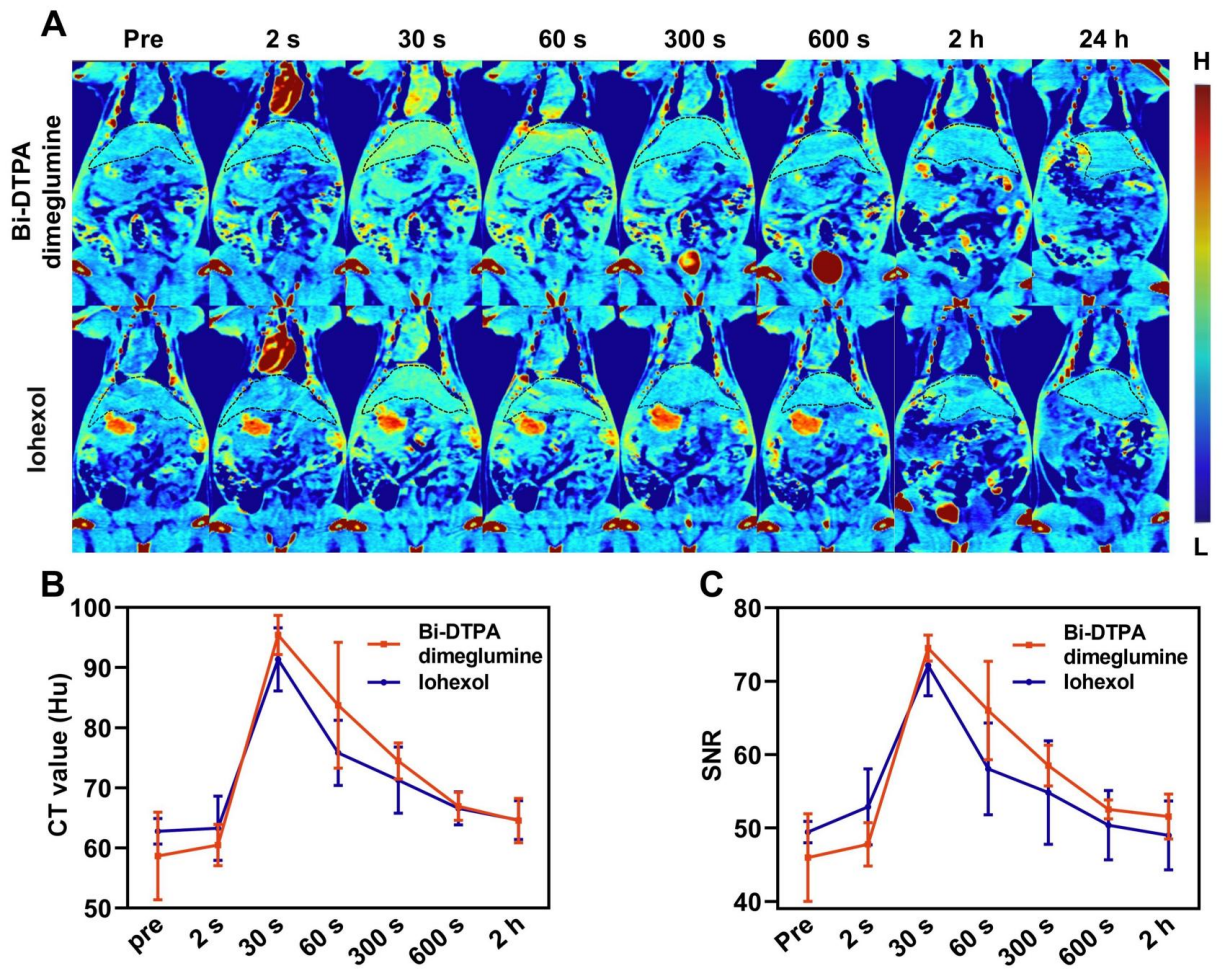
*In vivo* CT imaging of normal rats. After injecting doses of 334 mg Bi or 203 mg I/kg (1.6 mmol radiopaque elements/kg) of Bi-DTPA dimeglumine and iohexol, pseudo-colored images at different time points (pre, 2 s, 30 s, 60 s, 300 s, 600 s, 2 h and 24 h) (**A**), changes in liver CT values (**B**) and changes in liver region image SNR (**C**) were observed.

The rats received an injection of Bi-DTPA dimeglumine or iohexol at a dose of 203 mg I and 334 mg Bi/kg (equivalent to 1.6 mmol radiopaque elements/kg) into their tail veins using a high-pressure injection pump. This was followed by CT imaging scans. After two seconds of injecting Bi-DTPA dimeglumine, there was a noticeable increase in the signal intensity of the ventricle and aortic arch (Figure 3A). Thirty seconds later, the signal intensity enhancement reached its peak in the liver region (Figure 3B), with the CT value of the liver increasing from around 58 Hu to approximately 95 Hu, indicating a growth rate of about 63.79% (Figure 3B). At the same time, there was also an approximate increase of about 60% in SNR (Figure 3C). After one minute, there was a slight decrease in CT value observed in the liver (Figure 3A), and after five minutes, some Bi-DTPA dimeglumine had already been metabolized from the liver to reach the bladder through kidney excretion (Supplementary Figure S7A, C and D). At 2 h, the contrast enhancement in the liver and kidneys had become very faint, and most of the Bi-DTPA dimeglumine had been metabolized from the kidneys, with a significant portion metabolized to the bladder or excreted through urine (Figure 3A and Supplementary Figure S7A and C). At 24 h, most of the Bi-DTPA dimeglumine had been cleared from the body (Supplementary Figure S7A, C and D). The above phenomenon was mainly due to the short circulation half-life (16.38 min) of Bi-DTPA dimeglumine (Supplementary Figure S8). In addition, ICP-OES analysis indicated that after 24 h, the Bi levels in other major organs were virtually nonexistent, with only a small amount remaining in the kidneys. After one week, the amount of Bi in the kidneys was extremely low (Supplementary Figure S9). These results suggested that Bi-DTPA dimeglumine can be rapidly excreted by the kidneys and did not accumulate in major organs. Compared to iohexol, Bi-DTPA dimeglumine exhibits similar metabolic pathways and comparable *in vivo* diagnostic performance (Figure 3 and Supplementary Figures S7 and S9).

The *in vivo* liver imaging effects of Bi-DTPA dimeglumine in rats did not fully leverage the superior CT imaging capability inherent to Bi-DTPA dimeglumine to iohexol. This was attributed to the low concentration of imaging probes in the liver and the limited density resolution of the imaging equipment although the CT imaging equipment in this study is one of the most advanced imaging equipment internationally. Consequently, the relatively minor difference in contrast enhancement effect between Bi-DTPA dimeglumine and iohexol could not be adequately demonstrated. However, this does not diminish the superior contrast enhancement capability of Bi-DTPA dimeglumine. With the continuous innovation of higher-density resolution CT imaging devices, such as the rapidly advancing photon-counting CT, Bi-DTPA dimeglumine is expected to exhibit stronger imaging capabilities *in vivo* compared to iohexol. Iodine contrast agents commonly used in clinical practice pose issues related to iodine allergies [72] and contraindications [73], which prevent patients with allergic reactions or hyperthyroidism from undergoing enhanced CT examinations, significantly complicating the diagnosis of various diseases. In contrast, Bi-DTPA dimeglumine, as an iodine-free contrast agent, offers a viable alternative for these special populations. Therefore, the proposed Bi-DTPA dimeglumine-enhanced CT imaging method represents a promising alternative to traditional iodine contrast imaging, particularly in cases where iodine contrast agents are contraindicated.

### *In vivo* CT imaging of HIRI rats

**Figure 4.**
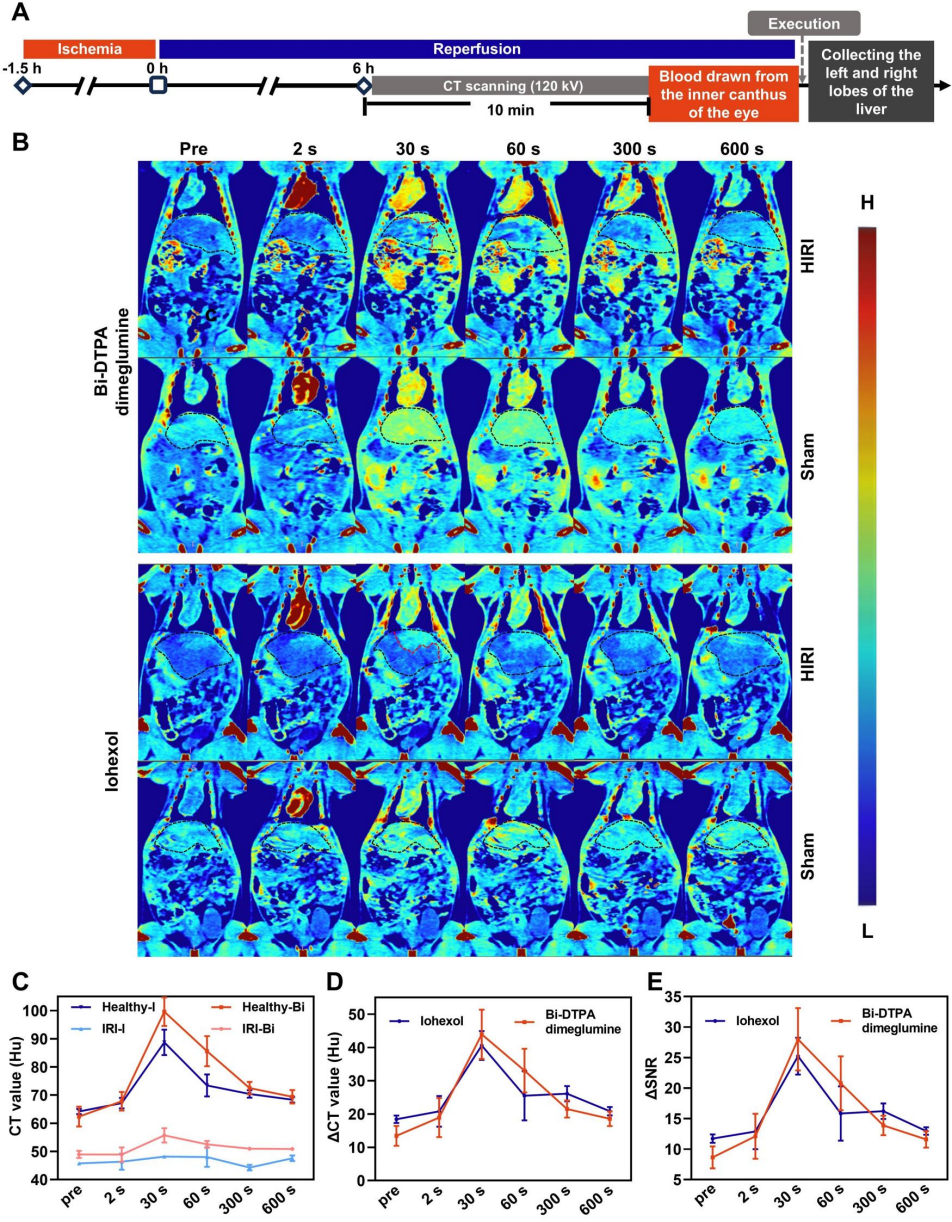
Contrast-enhanced CT imaging for liver ischemia for 1.5 h followed by reperfusion for 6 h. Construction of the CT scan model and flowchart (**A**). After injecting doses of 334 mg Bi or 203 mg I/kg (1.6 mmol radiopaque elements/kg) of Bi-DTPA dimeglumine and iohexol, pseudo-colored images at different time points (pre, 2 s, 30 s, 60 s, 300 s and 600 s) for the 6 h ischemia–reperfusion group and the sham surgery group are shown, and the dashed line outlined the entire liver and the boundary between the injured and healthy areas on the CT image (**B**). Changes in CT values of the injured and healthy areas before and after enhancement (**C**). ΔCT value (**D**) and ΔSNR (**E**) between the injured and healthy area images.

To investigate the imaging efficacy of Bi-DTPA dimeglumine in HIRI, we established a model of varying degrees of injury involving 70% ischemia–reperfusion in the left median lobes of the liver. Subsequently, Bi-DTPA dimeglumine or iohexol (1.6 mmol radiopaque elements/kg) was injected into the tail veins of anesthetized HIRI rats with a high-pressure injector at a rate of 0.5 mL/s (Figure 4A). Before injecting the imaging probe, the CT values in the liver injury regions (left and median lobes) were slightly lower than those in the healthy regions (right and caudate lobes) (Figure 4B), with a difference of approximately 14 Hu (48.92 ± 1.28 Hu vs 62.35 ± 3.52 Hu) (Figure 4C), in HIRI rats that underwent 1.5 h of ischemia followed by 6 h of reperfusion. However, the contrast between the two regions was insufficient, and the boundary of the liver injury area was indistinct (Figure 4B), making it impossible to accurately determine the location and extent of the injured sites. After the injection of Bi-DTPA dimeglumine, as time progresses, the CT value difference between the liver injury region and the healthy region gradually became more significant. The maximum difference occurs at 30 s post-injection (Figure 4D), where the signal enhancement in the injury region was very subtle, with the CT enhancement absolute value being less than 10 Hu (from 48.92 ± 1.28 to 55.69 ± 2.56 Hu) (Figure 4C). In contrast, the signal enhancement value in the healthy region exceeded 30 Hu (from 62.35 ± 3.52 to 99.64 ± 5.08 Hu) (Figure 4C). In addition, the ΔSNR increased from 8.65 ± 1.81 to 27.99 ± 5.13 (Figure 4E), which indicated the obviously enhanced contrast resolution after the injection of the imaging probe. After 30 s, as Bi-DTPA dimeglumine was gradually metabolized into the kidney, the CT value difference between the injury region and the healthy region decreased (Figure 4B). About 10 min later, blood was collected from the inner canthus of the rats, and the biochemical analysis results indicated that the rats subjected to CT scans did indeed experience liver injury based on significantly increased levels of ALT and AST (Supplementary Figure S10). Subsequently, the rats were sacrificed, and the healthy and injured liver lobes in each rat were then dissected for histopathological analysis, which indicated noticeable hepatic necrosis and tissue degeneration in the injured lobe, accompanied by inflammatory infiltration, while not in healthy lobe (Supplementary Figure S11), aligning with the findings from CT imaging (Figure 4B). To compare the diagnostic effects of iohexol and Bi-DTPA dimeglumine, HIRI rats were administered with iohexol (1.6 mmol I/kg) through the tail vein of rats for CT imaging (Figure 4B), and the results showed Bi-DTPA dimeglumine and iohexol exhibited the comparable imaging performance in HIRI rats.

**Figure 5.**
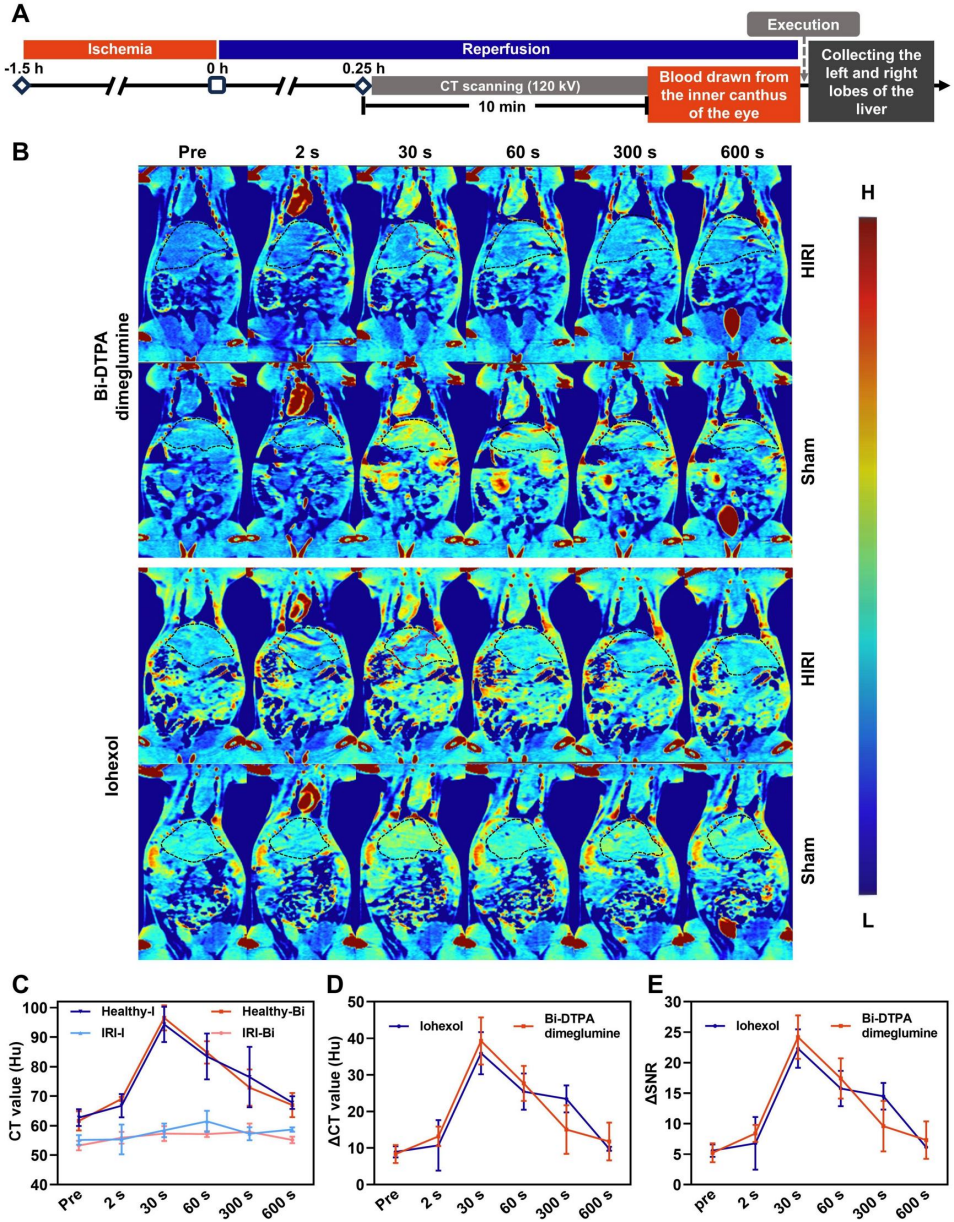
Contrast-enhanced CT imaging for liver ischemia for 1.5 h followed by reperfusion for 0.25 h. Construction of the CT scan model and flowchart (**A**). After injecting doses of 203 mg I or 334 mg Bi/kg (1.6 mmol I and Bi/kg) of Bi-DTPA dimeglumine or iohexol, pseudo-colored images at different time points (pre, 2 s, 30 s, 60 s, 300 s and 600 s) for the 0.25 h ischemia–reperfusion group and the sham surgery group are shown, and the dashed line outlined the entire liver and the boundary between the injured and healthy areas on the CT image (**B**). Changes in CT values of the injured and healthy areas before and after enhancement (**C**). ΔCT value (**D**) and ΔSNR (**E**) between the injured and healthy area images.

To further investigate the diagnostic potential of Bi-DTPA dimeglumine in HIRI with different injured degrees, we conducted CT imaging on rats subjected to 1.5 h of ischemia followed by 0.25 h of reperfusion (Figure 5A). Before the injection of the imaging probe, the CT values in the liver injury region were slightly lower than those in the healthy region (53.48 ± 1.63 Hu vs. 61.61 ± 3.25 Hu) (Figure 5B and C), and the boundary of the liver injury area was slightly indistinct. After the injection of Bi-DTPA dimeglumine, the CT value difference between the liver injury region and the healthy region gradually became significant. The maximum contrast occurred at 30 s post-injection (Figure 5D), when the ΔSNR between the injury region and healthy region peaked, increasing from 5.22 ± 1.55 to 24.19 ± 3.56 (Figure 5E). The subsequent blood testing and HE staining demonstrated the injury status of HIRI rats (Supplementary Figures S10 and S11), which was consistent of the CT imaging (Figure 5B). The levels of key indicators (AST, ALT) of rats subjected to 1.5 h of ischemia followed by 0.25 h (15 min) of reperfusion was lower than that subjected to 6 h of reperfusion which indicated the different injury degrees (Supplementary Figure S10). Iohexol-based CT imaging in HIRI rats revealed that Bi-DTPA dimeglumine and iohexol owned the similar imaging performance (Figure 5B–E). These results proved that Bi-DTPA dimeglumine as a non-iodine-based imaging probe enabled precise and fast diagnosis at different stages of HIRI.

## Conclusion

In summary, we proposed the use of non-iodine imaging probe, renal-clearable Bi-DTPA dimeglumine, for non-invasive fast evaluation of HIRI through CT imaging *in vivo*. The Bi-DTPA dimeglumine not only owned the advantages of simple synthesis process, no purification requirement, large-scale production capability, but also exhibited good biocompatibility, and outstanding CT imaging performance. For normal rats, the CT value of the liver can significantly increase by 63.79% in a short time (30 s) after the administration of Bi-DTPA dimeglumine. In the HIRI rat model, Bi-DTPA dimeglumine facilitated the clear and rapid differentiation between healthy and injured liver regions of various degrees based on notable differences in CT values, consistent with histopathological analysis findings. Additionally, Bi-DTPA dimeglumine was capable of complete elimination from the body *via* the renal pathway within 24 h. Bi-DTPA dimeglumine demonstrates promising potential for liver disease diagnosis as a replacement for iodine-based imaging probes with inherent limitations.

## Supplementary Material

rbae118_Supplementary_Data
