# Supplementary material for: Non-invasive fast assessment of hepatic injury through computed tomography imaging with renal-clearable Bi-DTPA dimeglumine
Source: Regen Biomater. 2024 Oct 3;11:rbae118. doi: 10.1093/rb/rbae118 (PMC11467190; doi:10.1093/rb/rbae118)
Supplement: rbae118_Supplementary_Data [file rbae118_supplementary_data.docx]

**Supporting Information**

Non-invasive fast assessment of hepatic injury through computed tomography imaging with renal-clearable Bi-DTPA dimeglumine

Li Ma^a,1^, Jinbin Pan^a,1^, Gang Shu^c^, Haiyan Pan^a^, Jingang Li^d^, Dong Li^a,^ *, Shao-Kai Sun^b,^ *

*^a^ Department of Radiology, Tianjin Key Laboratory of Functional Imaging, Tianjin Medical University General Hospital, Tianjin, 300052, China.*

*^b^ School of Medical Imaging, Tianjin Key Laboratory of Functional Imaging, Tianjin Medical University, Tianjin 300203, China.*

*^c^ Department of Radiology, The Second Hospital of Tianjin Medical University, Tianjin 300211, China.*

*^d^ Taishan Vocational College of Nursing, Shandong 271000, China.*

***Corresponding authors:** E-mail: dr_lidong@163.com (Dong Li), shaokaisun@tmu.edu.cn (Shao-Kai Sun)

**^1^ These authors contribute equally to this work.**

**Keywords:** Bismuth, Computed tomography, Hepatic ischemia reperfusion, Renal-clearable


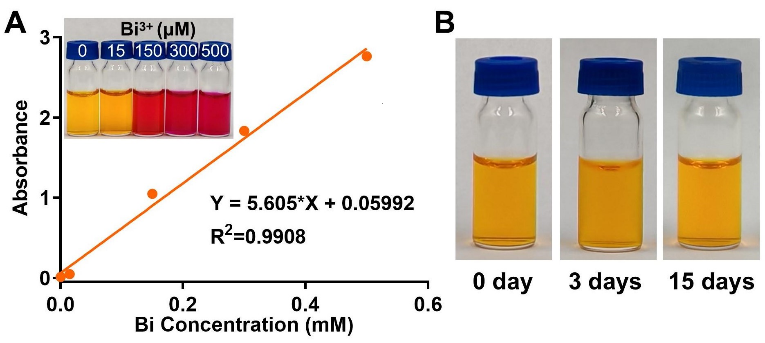


**Figure S1.** The stability and leakage of Bi-DTPA dimeglumine. (A) Standard curve for the quantitative determination of free Bi³⁺ concentration at 570 nm based on the formation of the red complex (Bi^3+^-XO). (B) Evaluation of Bi³⁺ leakage from Bi-DTPA dimeglumine (100 mM) over time.

**.
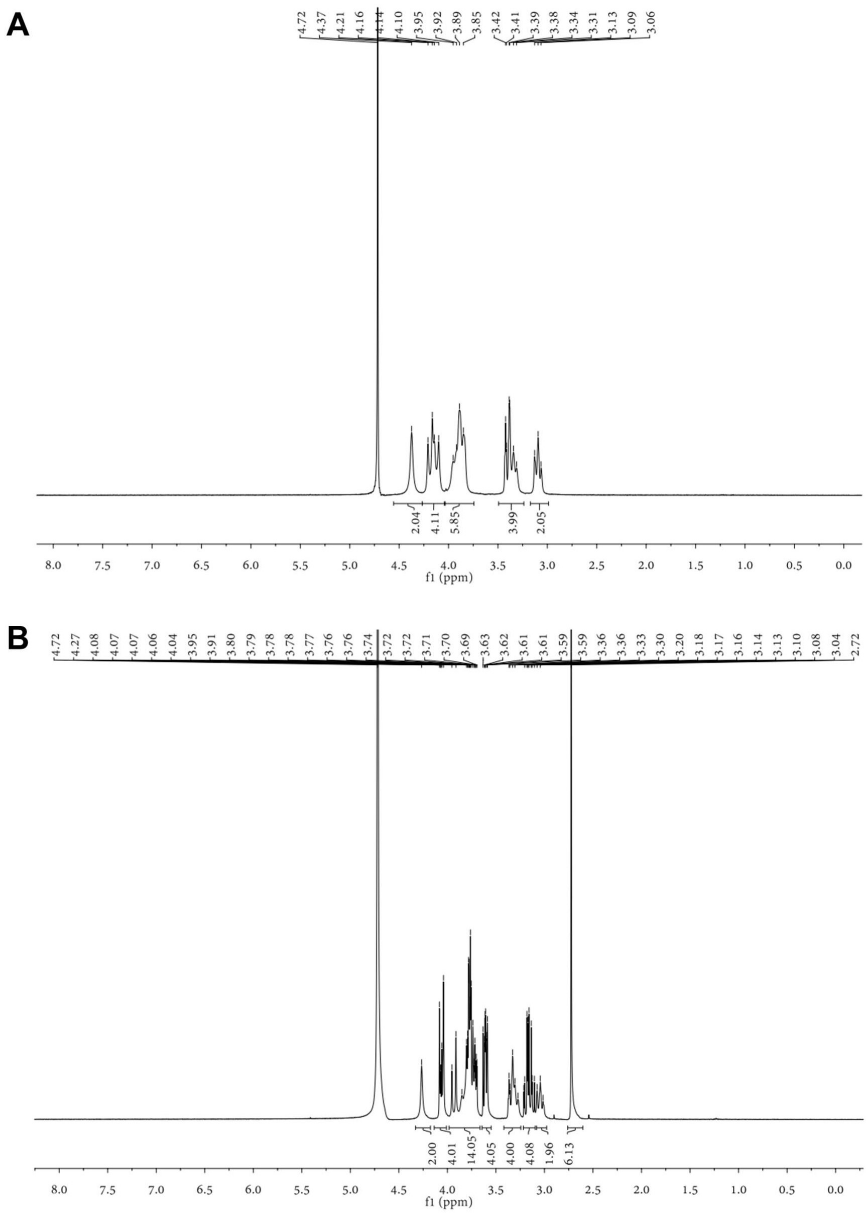
**

**Figure S2.** (A) ^1^H NMR spectrum of Bi-DTPA.^1^H NMR (400 MHz, D_2_O) δ 4.37 (s, 2H), 4.24 – 4.05 (m, 4H), 3.99 – 3.76 (m, 6H), 3.50 – 3.23 (m, 4H), 3.10 (t, J = 13.1 Hz, 2H). (B) ^1^H NMR spectrum of Bi-DTPA dimeglumine. ^1^H NMR (400 MHz, D_2_O) δ 4.27 (s, 2H), 4.13 - 4.01 (m, 4H), 3.97 - 3.67 (m, 14H), 3.66 - 3.56 (m, 4H), 3.41 - 3.25 (m, 4H), 3.22 - 3.09 (m, 4H), 3.04 (t, J = 12.3 Hz, 2H), 2.72 (s, 6H).


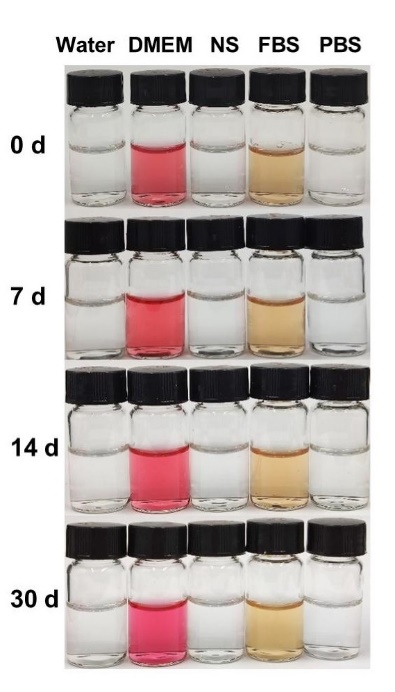


**Figure S3.** The long-term colloidal stability of Bi-DTPA dimeglumine in different media (from left to right: water, DMEM, NS, FBS, and PBS) at 0 day, 7 days, 14 days, and 30 days.


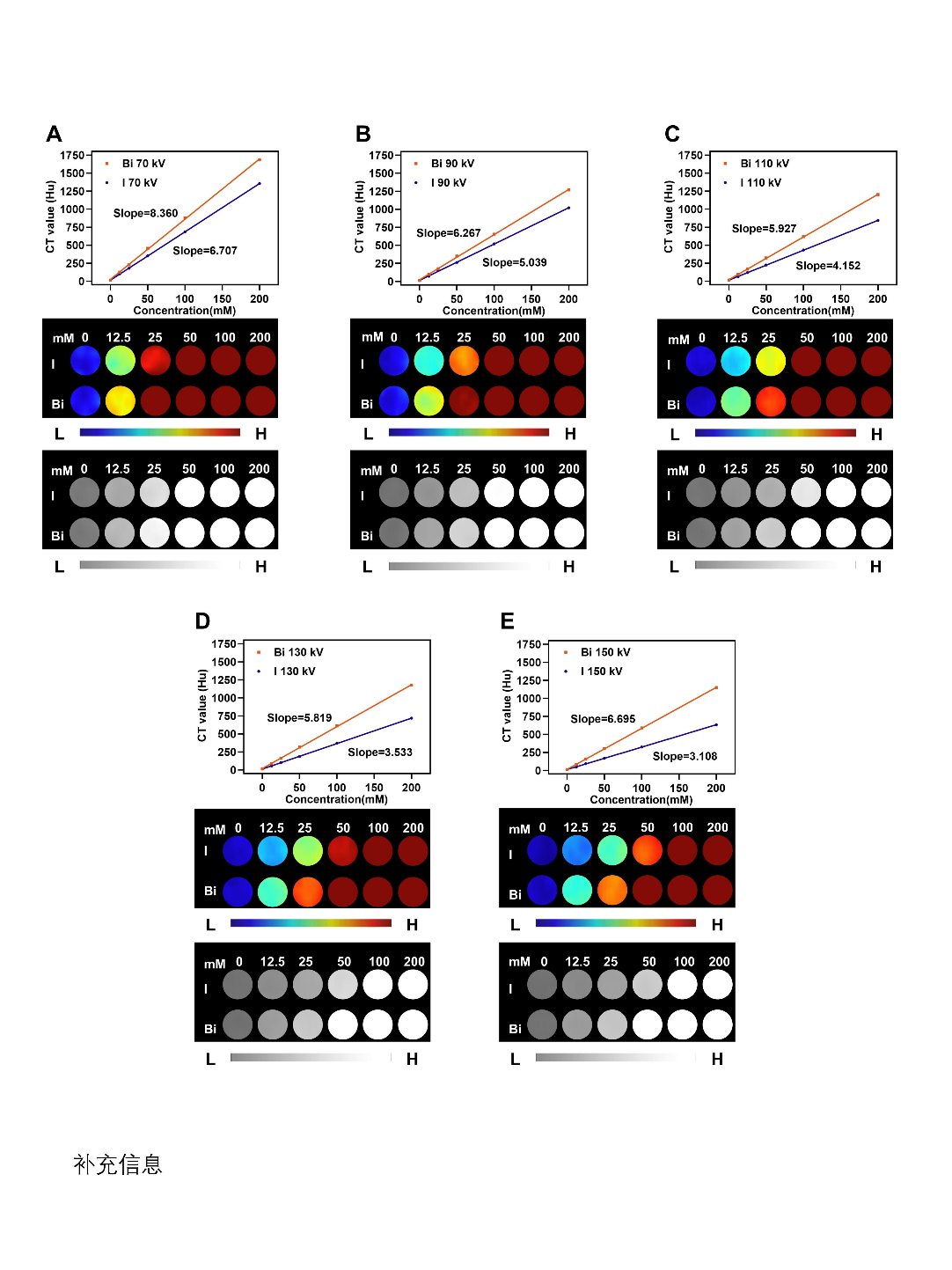


**Figure S4.** Different concentrations (0, 12.5, 25, 50, 100 and 200 mM Bi/I) of Bi-DTPA dimeglumine and iohexol under tube voltages of 70 kV (A), 90 kV (B), 110 kV (C), 130 kV (D), and 150 kV (E) in CT signal curves, CT pseudo-color, and grayscale images.


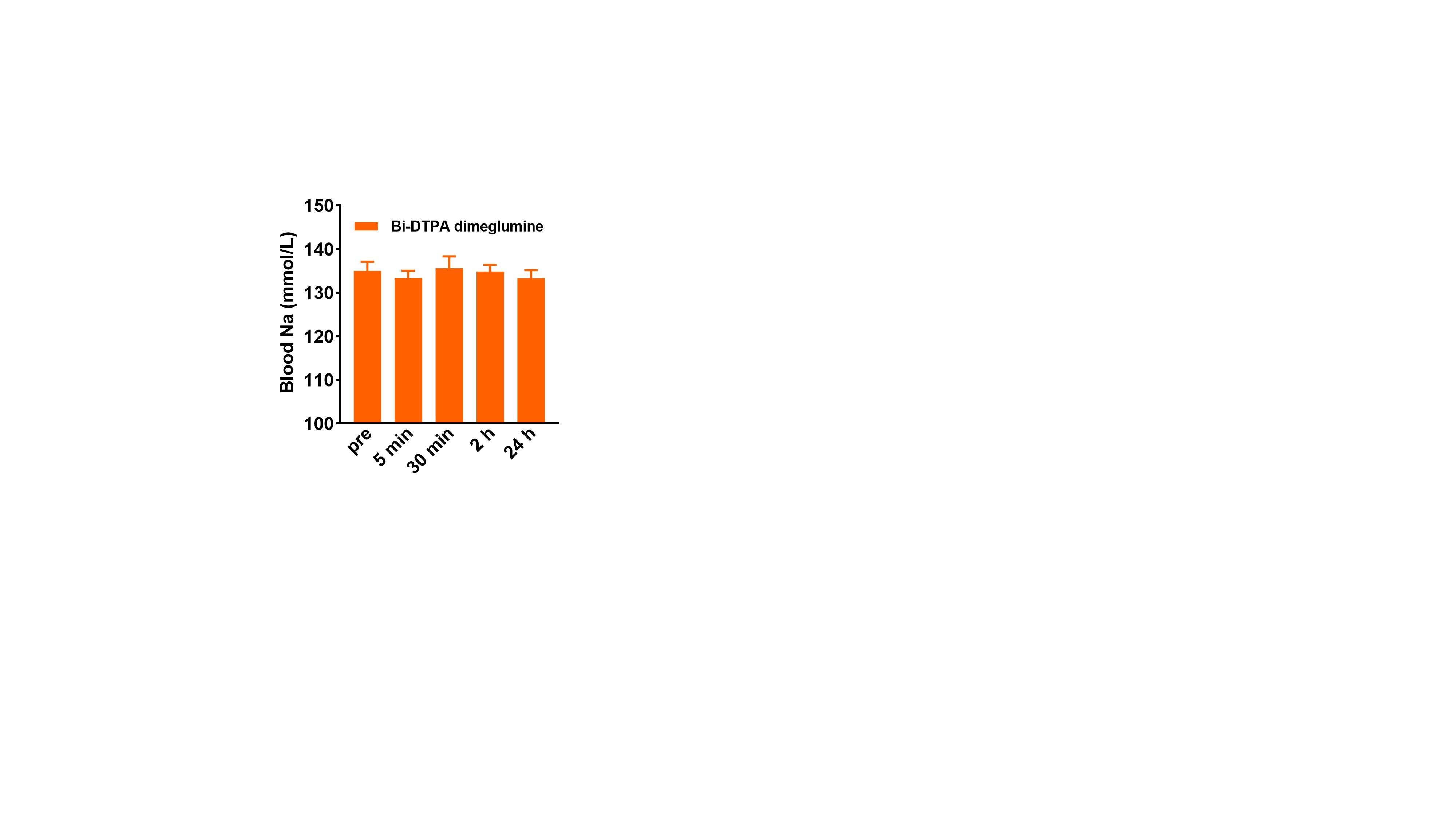


**Figure S5.** Blood Na level (mmol/L) of rats after injection of Bi-DTPA dimeglumine at different time points.


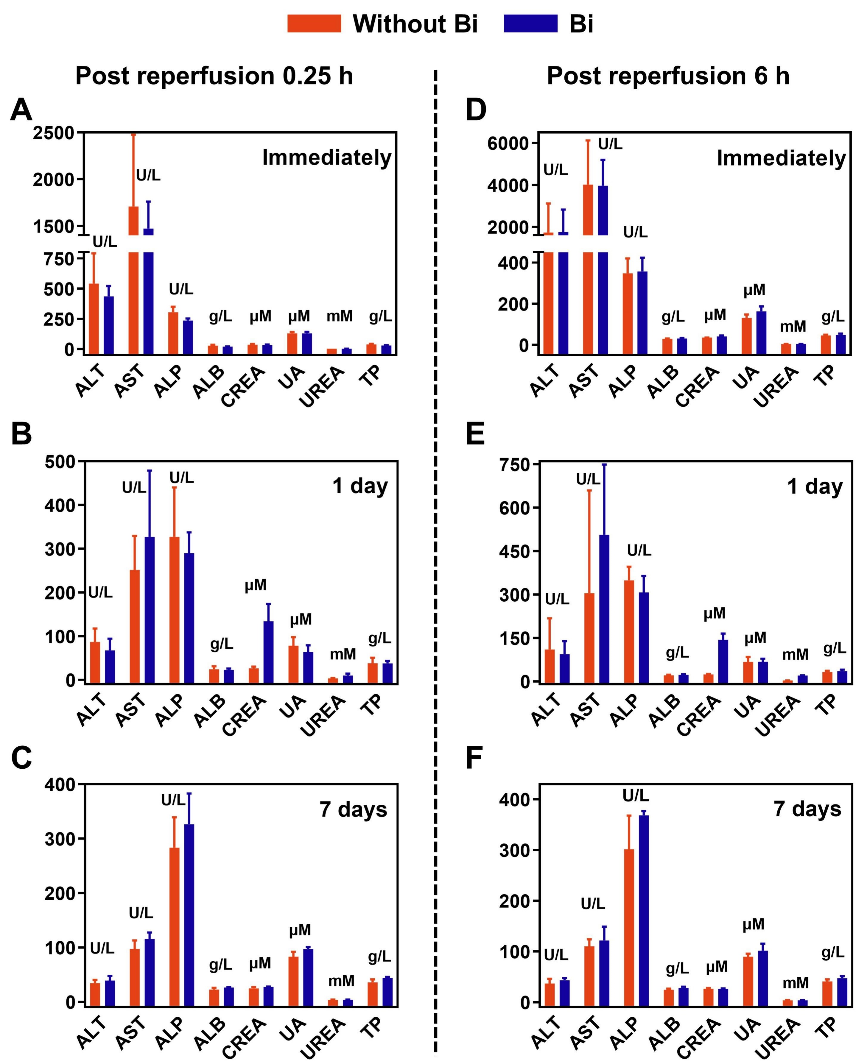


**Figure S6.** Rats with HIRI received either a 1.6 mmol Bi/kg dose of Bi-DTPA dimeglumine injection or no injection. Biochemical analyses of liver and kidney function were performed immediately, at 1 day, and at 7 days post-treatment.


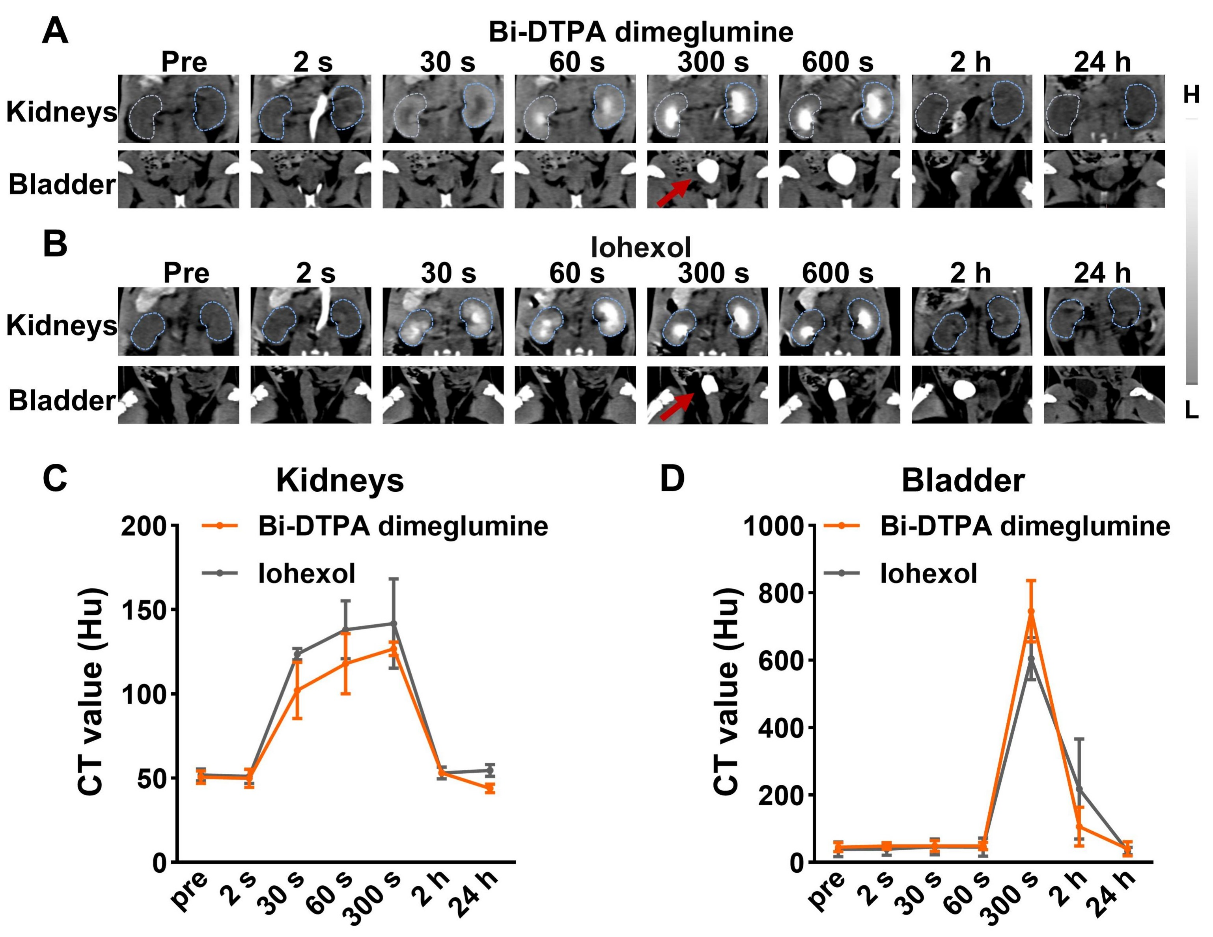


**Figure S7.** The in vivo metabolic behavior of imaging probes was assessed through CT scans of the kidneys and bladder at 0 s, 2 s, 30 s, 60 s, 300 s, 600 s, 2 h, and 24 h after the injection of Bi-DTPA dimeglumine (A) or iohexol (B), and the red arrow pointed to the bladder. CT value curves of kidneys (C) and bladder (D) after injection of Bi-DTPA dimeglumine and iohexol.


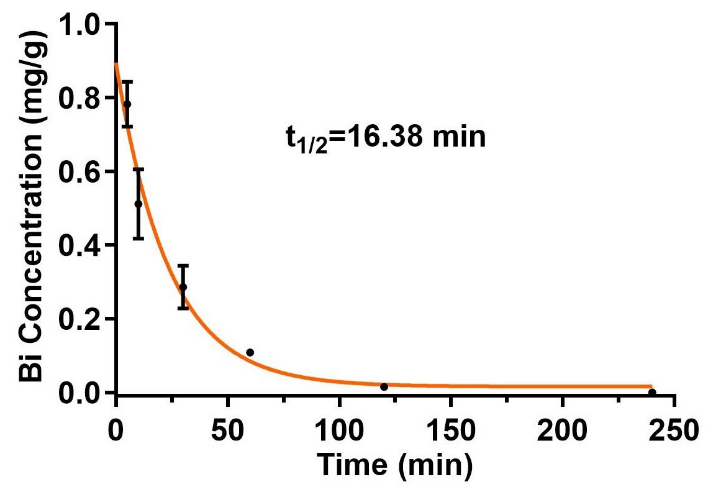


**Figure S8.** The circulation half-life of Bi-DTPA dimeglumine.


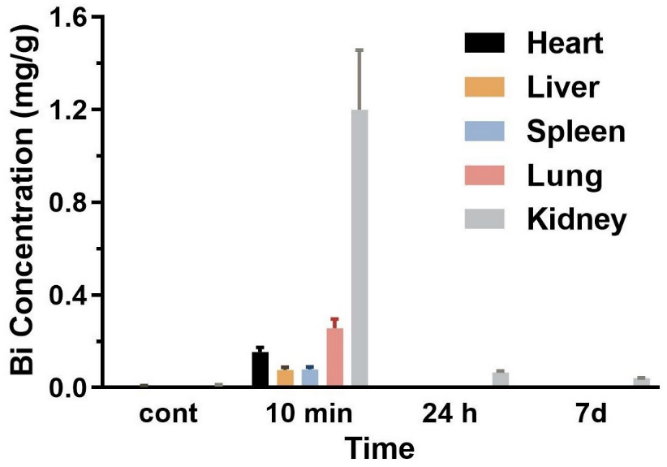


**Figure S9.** Biodistribution of Bi-DTPA dimeglumine in various organs following intravenous administration.

**
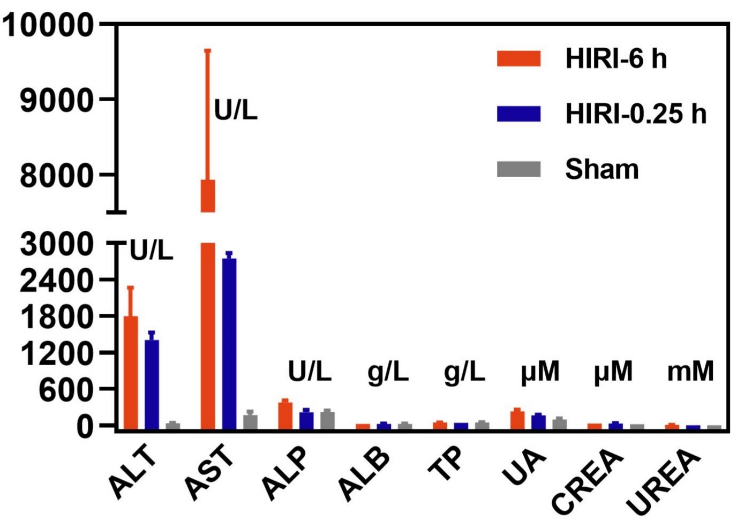
**

**Figure S10.** Comparison of hepatic and renal biochemical indicators at different reperfusion times (6 h, 0.25 h) with the sham surgery group (n=3).


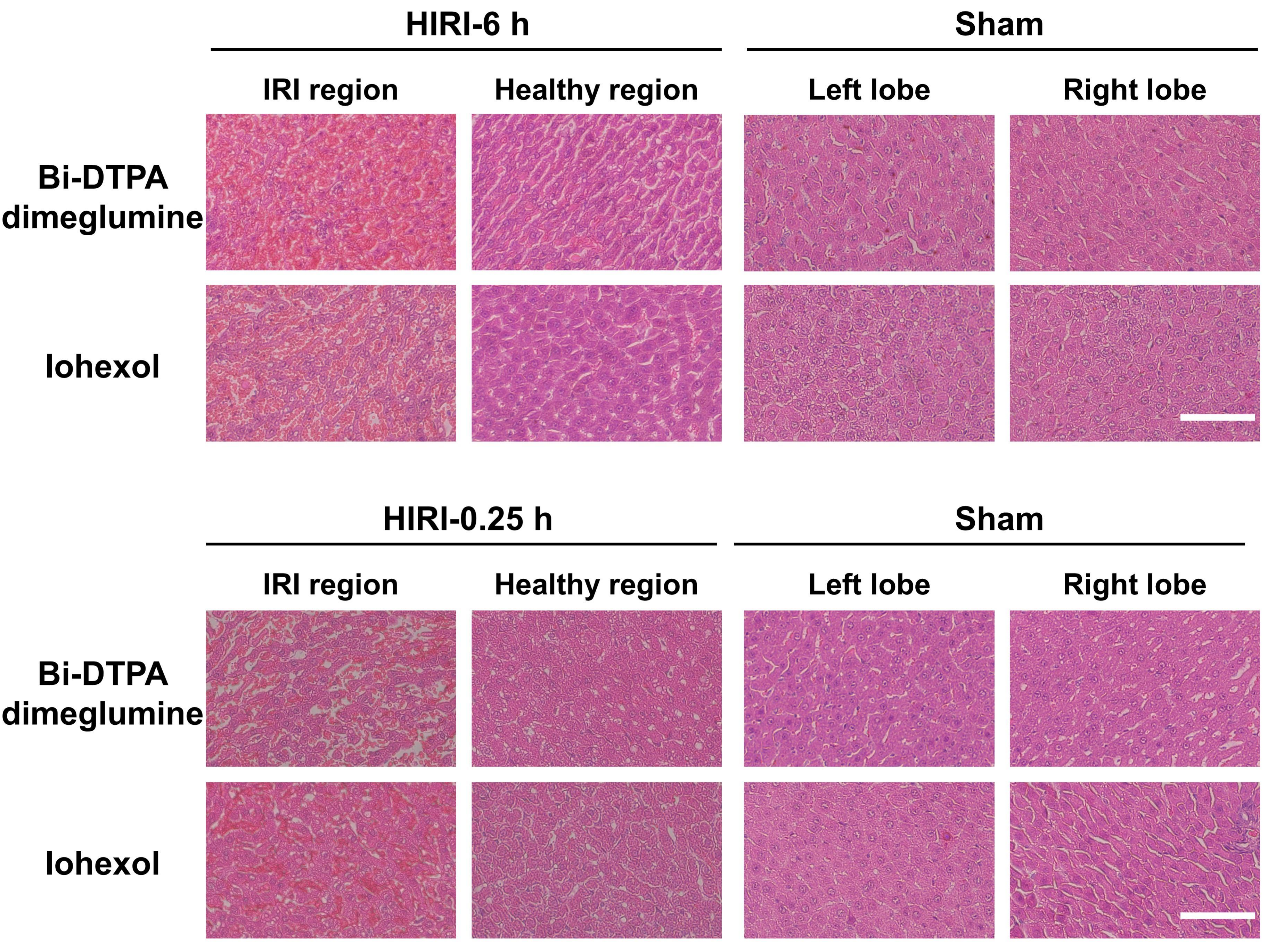


**Figure S11.** Pathological analysis of different liver regions in rats (with varying degrees of liver injury and sham surgery group) undergoing CT-enhanced imaging using Bi-DTPA dimeglumine or iohexol. Scale bar: 100 μm.
